# Dietary Squalene Increases High Density Lipoprotein-Cholesterol and Paraoxonase 1 and Decreases Oxidative Stress in Mice

**DOI:** 10.1371/journal.pone.0104224

**Published:** 2014-08-12

**Authors:** Clara Gabás-Rivera, Cristina Barranquero, Roberto Martínez-Beamonte, María A. Navarro, Joaquín C. Surra, Jesús Osada

**Affiliations:** 1 Departamento Bioquímica y Biología Molecular y Celular, Facultad de Veterinaria, Instituto de Investigación Sanitaria de Aragón (IIS), Universidad de Zaragoza, Zaragoza, Spain; 2 Departamento de Producción Animal, Escuela Politécnica Superior de Huesca, Huesca, Spain; 3 CIBER de Fisiopatología de la Obesidad y Nutrición, Instituto de Salud Carlos III, Madrid, Spain; Heart Research Institute, Australia

## Abstract

**Background and Purpose:**

Squalene, the main hydrocarbon in the unsaponifiable fraction of virgin olive oil, is involved in cholesterol synthesis and it has been reported to own antiatherosclerotic and antiesteatosic effects. However, the squalene's role on lipid plasma parameters and the influence of genotype on this effect need to be addressed.

**Experimental Approaches:**

Three male mouse models (wild-type, *Apoa1-* and *Apoe-* deficient) were fed chow semisynthetic diets enriched in squalene to provide a dose of 1 g/kg during 11 weeks. After this period, their plasma parameters and lipoprotein profiles were analyzed.

**Key Results:**

Squalene administration at a dose of 1 g/kg showed decreased reactive oxygen species in lipoprotein fractions independently of the animal background and caused an specific increase in high density lipoprotein (HDL)-cholesterol levels, accompanied by an increase in phosphatidylcholine and paraoxonase 1 and no changes in apolipoproteins A1 and A4 in wild-type mice. In these mice, the cholesterol increase was due to its esterified form and associated with an increased hepatic expression of *Lcat*. These effects were not observed in absence of apolipoprotein A1. The increases in HDL- paraoxonase 1 were translated into decreased plasma malondialdehyde levels depending on the presence of Apolipoprotein A1.

**Conclusions and Implications:**

Dietary squalene promotes changes in HDL- cholesterol and paraoxonase 1 and decreases reactive oxygen species in lipoproteins and plasma malondialdehyde levels, providing new benefits of its intake that might contribute to explain the properties of virgin olive oil, although the phenotype related to apolipoproteins A1 and E may be particularly relevant.

## Introduction

Virgin olive oil is the main source of fat in the Mediterranean dietary pattern, and it was shown an important relationship between olive oil intake and the reduced cardiovascular risk [1], [2], [3], and even cardiovascular mortality [4]. The mechanism of this protective effect of virgin olive oil intake still needs to be elucidated. It was firstly attributed to its main components, monounsaturated fatty acids, and especially to the most abundant, oleic acid. However, the interest has been lately focused on its minor bioactive components since biological actions of these compounds have been documented [2], [5]–[7].

Squalene, first isolated from shark liver oil and named by Tsujimoto in 1916 [8], is a polyunsaturated triterpene containing six isoprene units and a biochemical precursor of cholesterol and other steroids [9]. Squalene content in extra virgin olive oil is especially high, up to 0.7% (7 g/kg), compared to other oils and human dietary fats [10], [11]. *In vitro*, it is a highly effective oxygen scavenging agent [12] and stable in virgin olive oil heated at 180°C for 36 h [13]. For these reasons, it has been even stated that squalene rather than oleic acid could be the important active compound of virgin olive oil [14].

In humans, orally administered squalene is well absorbed (60–85%). This and the intestinal *de novo* synthesized squalene are transported by chylomicrons into circulation, being rapidly taken up by the liver, where it is converted into sterols and bile acids [15] or secreted into bloodstream [16], [17]. Hepatic squalene, either biosynthesized or dietary, is secreted into very low density lipoproteins (VLDL) and low density lipoproteins (LDL) and distributed to various tissues. Skin is also a biosynthetic tissue for squalene to provide the large quantities found in the sebaceous glands [18], [19]. Therefore, squalene concentration in plasma lipoproteins represents an equilibrium from dietary intake, with important amounts coming from extra virgin olive oil, and intestinal or liver synthesis [20].

Nonetheless, it is not clear yet squalene's role on plasma lipids in humans and animal models. While some authors did observe increased plasma cholesterol after squalene intake in rats [21] and in hamsters [22], others did not in humans [23], neither in rats [24] nor in *Apoe*-deficient mice [25]. These discrepancies raise the questions of whether animal models or administration regimens (length and doses) are modifying squalene's effect on plasma cholesterol. Another aspect requiring attention is the long-term effects and safety of high dose of squalene consumption [26].

Mice are considered high density lipoprotein (HDL) animals, since they lack cholesteryl ester transfer protein and most of their plasma cholesterol is transported in these lipoproteins [27]. Among mouse strains, C57BL/6J genetic background is widely used due its higher predisposition to atherosclerosis development [28]. Moreover, mice have been subjects of intensive genetic manipulation, and genes codifying for proteins associated with HDL have been created and tested to study their biological functions [29]. Indeed, mice lacking apolipoprotein A1 (APOA1), the main component of HDL, offer the possibility to explore changes in absence of HDL-containing APOA1 [30]. *Apoe*-deficient mice lack HDL-containing APOE, hence no apolipoprotein E can be transferred to remnant chylomicrons, and the latter are not taken up by the liver [31]. Both *Apoa1*- and *Apoe*-deficient mice are also available on C57BL/6J genetic background. Therefore, these models provide interesting experimental approaches to analyze the specific influence of these genotypes on the observed outcome following the long term administration of a semipurified diet supplemented with squalene. To this end, plasma parameters and lipoprotein profiles, gene expression and oxidative parameters will be analyzed in these three mouse models.

## Results

### Body and liver weight

Animal somatometric variables are shown in Table 1. In the studied models, the administration of dietary squalene had no effect on body weight, body weight gain or feed intake (data not shown). Besides, no significant changes were observed for liver weight, with the exception of a slight significant increase of this parameter in WT mice consuming the diet enriched in squalene that was not translated into changes of hepatic fat content measured as lipid droplet extent (data not shown). These results suggest that long term squalene administration is well tolerated in terms of body weight changes.

**Table 1:** Effect of the experimental diets on somatic variables in male mice of the three experimental models.

	Final body weight (g)	Body weight gain (g)	Liver weight (g)
**Wild-type**			
Control (n = 6)	30±1	3.1±1.2	1.2±0.1
Squalene (1 g/kg) (n = 7)	30±2	3.6±2.2	1.3±0.1*
***Apoa1*** **-deficient**			
Control (n = 7)	29±4	4.8±5.0	1.0±0.3
Squalene (1 g/kg) (n = 7)	27±3	2.8±5.1	1.0±0.2
***Apoe*** **-deficient**			
Control (n = 13)	35±2	5.5±2.2	1.8±0.9
Squalene (0.25 g/kg) (n = 13)	34±3	4.7±2.4	1.6±0.3
Squalene (1 g/kg) (n = 14)	33±3	4.5±1.8	1.6±0.4

Results are shown as mean values with their standard deviations. Statistical analysis was carried out using Mann Whitney U test.

* p<0.05 *vs* control.

### Plasma parameters

The effects of squalene on plasma parameters in WT, *Apoa1-* and *Apoe*-deficient mice after the 11-week experimental period are shown in Tables 2, 3 and 4. There were no significant differences in plasma triglycerides, neither in total cholesterol when the 1 g/kg squalene dose was provided to any mouse model. Nevertheless, a dose of 0.25 g/kg squalene significantly increased total cholesterol levels in *Apoe*-deficient mice (Table 4). HDL-cholesterol levels were raised in all mouse models when they were administered the high squalene dose. The increased HDL-cholesterol in wild type was not accompanied by increases in APOA1, nor in APOA4. However, paraoxonase, estimated as arylesterase activity, was increased in WT and *Apoa1*-deficient mice (Tables 2 and 3). Wild-type and *Apoa1*-deficient mice consuming 1 g/kg squalene (Tables 2 and 3) also showed increased plasma glucose levels. Non-esterified fatty acids were increased in wild-type and *Apoe*-deficient mice consuming 1 g/kg and 0.25 g/kg of squalene, respectively.

**Table 2:** Effect of dietary squalene supplementation on plasma parameters in male wild-type mice.

	Control (n = 6)	Squalene 1 g/kg (n = 7)
Total cholesterol (mM)	5.9±0.7	6.5±0.8
HDL-cholesterol (mM)	2.6±0.5	3.1±0.4*
Triglycerides (mM)	1.8±0.5	1.6±0.4
Glucose (mM)	18±1	20±1*
Non-esterified fatty acids (mM)	0.8±0.1	1.0±0.2*
APOA1 (AU)	20±2	19±1
APOA4 (AU)	7±1	7±1
Arylesterase activity (×10^3^ IU/L)	63±6	69±3*

Results are shown as mean values with their standard deviations. Statistical analysis was carried out using Mann Whitney U test.

* p<0.05 *vs* control.

AU, arbitrary units.

**Table 3:** Effect of dietary squalene supplementation on plasma parameters in male *Apoa1*-deficient mice.

	Control (n = 7)	Squalene 1 g/kg (n = 7)
Total cholesterol (mM)	0.8±0.4	0.9±0.3
HDL-cholesterol (mM)	0.4±0.1	0.6±0.1*
Triglycerides (mM)	0.6±0.2	0.5±0.1
Glucose (mM)	20±2.0	22±0.6*
Non-esterified fatty acids (mM)	0.5±0.1	0.5±0.1
Arylesterase activity (×10^3^ IU/L)	48±3	51±2*

Results are shown as mean values with their standard deviations. Statistical analysis was carried out using Mann Whitney U test.

* p<0.05 *vs* control.

**Table 4:** Effect of dietary squalene supplementation on plasma parameters in male *Apoe*-deficient mice.

	Control (n = 13)	Squalene 0.25 g/kg (n = 13)	Squalene 1 g/kg (n = 14)
Total cholesterol (mM)	23±6	28±2**	21±8∧∧
HDL-cholesterol (mM)	0.9±0.2	0.8±0.3	1.1±0.2* ∧
Triglycerides (mM)	3.4±1.3	3.7±0.9	3.4±1.4
Glucose (mM)	22±3	24±3	21±3∧
Non-esterified fatty acids (mM)	1.5±0.3	1.7±0.3**	1.4±0.6
Arylesterase activity (×10^3^ IU/L)	77±6	78±5	78±4

Results are shown as mean values with their standard deviations. Statistical analysis was carried out using Mann Whitney U test.

*p<0.05,

**p<0.01 mean values were significantly different from those of the control diet, and

∧ p<0.05,

∧∧ p<0.01 mean values were significantly different between 0.25 and 1 g/kg squalene doses.

### Effect of squalene on lipoprotein profiles

**Figure 1:**
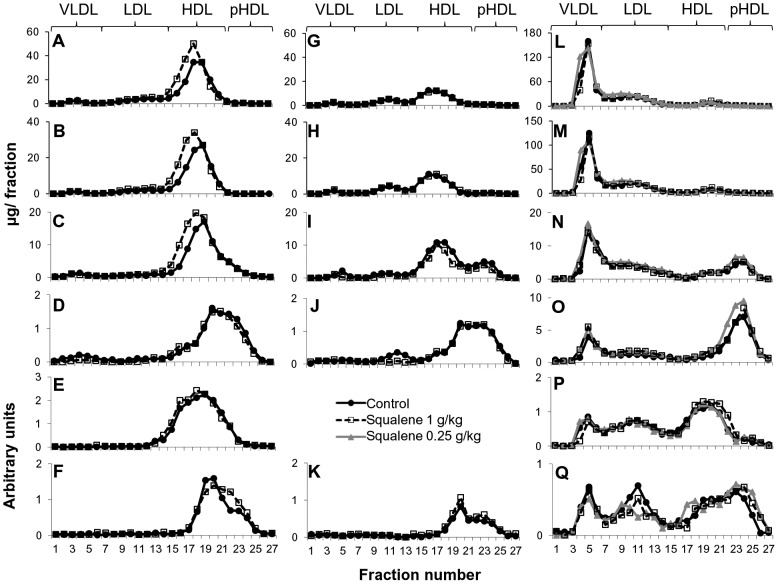
Effect of squalene on lipoproteins from the three experimental models. Plasma was obtained following 11 weeks of consuming control or squalene- enriched semipurified diets and after a four-hour fast. Two independent pools of all mice per experimental group were prepared, except for *Apoe*-deficient mice, where three plasma pools were utilized. Lipoproteins were separated by FPLC, and collected fractions analyzed for total cholesterol (A, G, L), esterified cholesterol (B, H, M), phosphatidylcholine (C, I, N), sphingomyelin (D, J, O), APOA1 (E, P) and APOA4 (F, K, Q). Representative profiles are shown from WT mice, left panels (control and squalene pools consisting of plasma from 6 and 7 mice, respectively), *Apoa1*-deficient mice, middle panels (n = 7 for control and n = 7 for squalene plasma pool) and Apoe-deficient mice, right panels (n = 13 for control, n = 13 for 0.25 g/kg and n = 14 for 1 g/kg squalene plasma pool). Fraction numbers 1–6 corresponded to VLDL/chylomicron remnants, 7–13 to low density lipoproteins, 14–21 to cholesterol-rich HDL and 22–27 to cholesterol-poor HDL (pHDL).

The distribution of the plasma parameters in lipoprotein fractions separated by FPLC from the different mouse models is shown in Figure 1. According to the model, cholesterol distribution changed. In this regard, cholesterol was mainly carried in HDL in WT mice (Fig. 1A), lower cholesterol levels in the latter lipoproteins and some cholesterol in VLDL and LDL fractions were observed in *Apoa1*-deficient mice (Fig. 1G), and the plasma cholesterol of *Apoe*-deficient mice was mainly accumulated in VLDL fraction (Fig. 1L). In wild-type mice, squalene administration induced an increase in HDL cholesterol (Fig. 1A), mainly due to esterified cholesterol (Fig. 1B), that was accompanied by increased HDL phosphatidylcholine (Fig. 1C), whilst no changes in sphingomyelin and APOA1, and redistribution of APOA4 towards smaller HDL particles were observed. In *Apoa1*-deficient mice, squalene did not have any effect on cholesterol distribution (Fig. 1G and H), decreased phosphatidylcholine (Fig. 1I) and increased APOA4 in HDL particles (Fig. 1K) and did not modify HDL sphingomyelin but decreased this phospholipid in LDL (Fig. 1J). In *Apoe*-deficient mice, squalene administration at either studied dose had little effect on cholesterol, esterified cholesterol, phosphatidylcholine and sphingomyelin (Fig. 1, panels L, M, N and O, respectively). However, it increased the presence of APOA4 in HDL and decreased LDL APOA4 (Fig. 1Q). Thus, these results indicate that squalene is modifying HDL composition depending on the presence of APOE and APOA1.

### Oxidative stress variables

**Figure 2:**
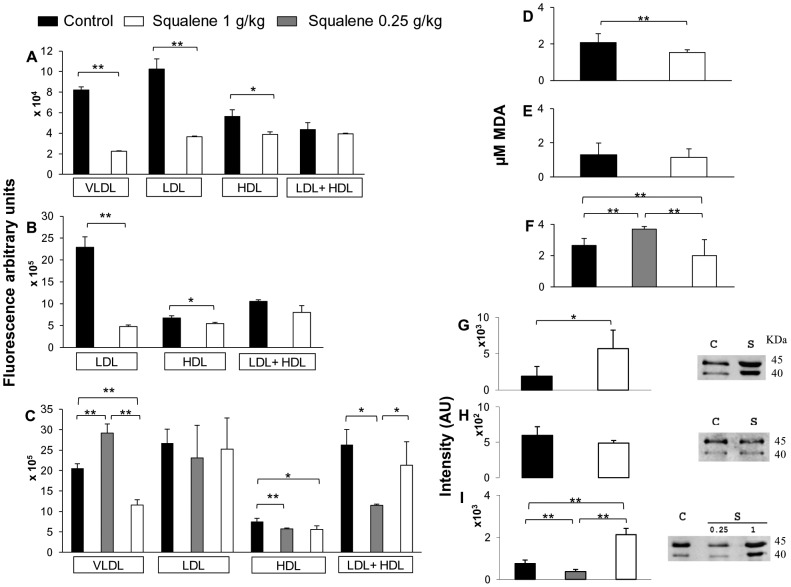
Effect of squalene on antioxidative parameters from the three experimental models. ROS levels in lipoprotein fractions from control and squalene treated mice expressed as arbitrary fluorescence units after incubation of lipoprotein fractions during 24 h with 2′,7′-dichlorofluorescein diacetate A) 0.8 µg LDL or VLDL, and 60 ng HDL from WT mice; B) 0.8 µg LDL and 60 ng HDL from *Apoa1*-deficient mice; C) 1.5 µg LDL or VLDL, and 60 ng HDL from *Apoe*-deficient mice. Each pool was assayed in triplicate. Individual plasma malondialdehyde levels from WT (D), *Apoa1*-(E) and *Apoe*-deficient mice (F). HDL-PON1 levels from WT (G), *Apoa1*-(H) and *Apoe*-deficient mice (I) with their representative Western blots. Each pool was assayed in triplicate. Results are shown as means ± SD. *p<0.05, **p<0.01 according to corrected unpaired t Welch's test.

As shown in Figures 2A and 2B, isolated LDL from the squalene-treated groups had significantly lower levels of reactive oxygen species than LDL from control animals in WT as well as in *Apoa1*-deficient mice. This effect was not observed in *Apoe*-deficient mice (Fig. 2C), although in this model receiving the high squalene dose, the ROS content in VLDL was significantly decreased as in wild-type. According to the modified HDL composition, a decrease in HDL-ROS levels was found in the three genotypes of mouse by the squalene administration, but there were no differences in the ability of HDL to inactivate LDL ROS species (Fig. 2A and B), except for *Apoe*-deficient mice, as LDL particles from WT and *Apoa1*-deficient squalene-receiving mice present already very low levels of ROS compared to control mice.

**Figure 3:**
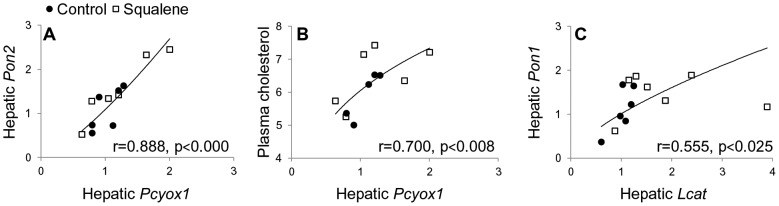
Relationship between hepatic gene expression and plasma cholesterol in wild type mice. Direct correlations among *Pcyox1* and *Pon2* gene expression (A), or plasma cholesterol (B), and *Lcat* and *Pon1* gene expression (C). Association analyses were carried out using Spearman's test for non- parametric distributions.

Figure 2 (panels D and F) shows significant decreases of plasma malondialdehyde (MDA) levels in WT and *Apoe*-deficient mice receiving squalene at 1 g/kg dose, what is an indicator of lower oxidative levels induced by squalene intake. No significant changes were seen in *Apoa1*-deficient mice (Fig. 2E). To corroborate whether the results of arylesterase activity corresponded to PON1, its HDL levels were determined. As shown in Figure 2 (panels G and I), mice administered squalene at 1 g/kg showed increased HDL-PON1 in WT and *Apoe*-deficient mice. No significant changes were observed in *Apoa1*-deficient mice (Fig. 2H). Since PON2 is a ubiquitous antioxidant enzyme [32], we investigated whether the squalene administration induced any differential hepatic mRNA expression in wild-type mice. According to results depicted in Table 5, hepatic *Pon2* expression was increased, so was *Pon1*. Furthermore, the hepatic mRNA expression of prenylcysteine oxydase 1, *Pcyox1* (a pro-oxidant enzyme of LDL [33]) was also tested (Table 5), and no significant change was observed either. Hepatic *Pcyox1* expression was found significantly associated with *Pon2* expression and with plasma cholesterol (Figure 3A and B). In contrast, hepatic *Lcat* expression was significantly increased (Table 5) and significantly associated with that of *Pon1* (Figure 3C).

**Table 5:** Effect of squalene on hepatic gene expression in male wild-type mice.

Gene	Control (n = 6)	Squalene (n = 7)
*Lcat*	1.0±0.2	1.4±0.7*
*Pon1*	1.1±0.5	1.5±0.4**
*Pon2*	1.1±0.5	1.4±0.6*
*Pcyox1*	1.0±0.3	1.2±0.5

Data (mean ± SD) represent arbitrary units normalized to the *Cyclophilin B*, *Cyclophilin A* and *Tbp* expressions for control and treated mice according to RT-qPCR. Statistical analysis was carried out by Mann-Whitney-U test,

*p<0.05,

** p<0.01.

## Discussion

The aims of present study were to verify the influence of genetic background and long-term administration of squalene on mouse plasma parameters and lipoprotein distribution in different mouse models. After 11-week administration, the main findings were that a high dose of squalene (1 g/kg mouse) increased HDL-cholesterol and PON1 levels with independence of the genetic background without increasing total cholesterol and thus decreasing total/HDL-cholesterol ratio. The increase in HDL-cholesterol was mainly due to increased esterified cholesterol and phosphatidylcholine, in absence of changes in APOA1 and APOA4 levels and correlated with arylesterase activity, PON1 levels and hepatic *Lcat* mRNA expression in wild-type mice. Furthermore, lipoprotein ROS content was diminished by squalene administration in function of genotype. Overall, squalene is an important modifier of HDL metabolism with important differences regarding genotype and dose.

The highest squalene dose used in the present study was 1 g/kg/day, which is five times lower than the lethal dose 50 (5 g/kg/day) [34] and far lower than the dose required to induce encephaloneuropathy in rats (20 g/kg/day) [35]. Considering the higher metabolic rate of mice [36], our dose would translate into a human of 100 mg/kg/day. This is clearly higher than the highest reported in human nutritional studies (15 mg/kg/day) [17], but lower than the pharmacological doses of 185 and 385 mg/kg/day provided to female volunteers for 90 days [37]. Therefore, the present study uses a dose higher than those provided in human nutritional interventions, but lower than the used in pharmacological trials.

HDL levels are an inverse risk factor for coronary artery diseases and the total/HDL-cholesterol ratio has been reported to be a better predictor of ischemic heart disease than other conventional risk markers (total cholesterol, LDL-cholesterol or triacylglycerides) [38]–[42]. In this way, a high dose of 1 g/kg of squalene could be protective since it raised HDL-cholesterol without modifying total cholesterol in all studied mouse models, confirming our previous study using this dose [25]. However, an increase in total cholesterol was observed at a low squalene dose (0.25 g/kg mouse) in *Apoe*-deficient mice without increasing HDL-cholesterol. In this regard, discrepant effects of squalene on plasma lipids have been previously reported. Indeed, a lowering plasma cholesterol effect was described in rats using a 1 g/kg dose during 4 weeks [24] and the opposite was also found in this animal consuming the same dose in corn oil [21]. In humans, dietary supplementation of squalene at 12 mg/kg formulated in rapeseed oil for nine weeks was reported to cause increases in total and LDL-cholesterol concentrations [16] or not to have effect when provided as pills [23]. However, a subsequent six-week period on a lower dose of 6 mg/kg normalized serum sterols [43]. Low dose had a hypercholesterolemic effect in hamsters [22] as well. This discrepant outcome shows that squalene effect on plasma cholesterol is dose dependent, as low doses seem to promote cholesterol synthesis (squalene is an intermediary in cholesterol biosynthesis), and high doses might have a different effect. A potential action could be related to an intestinal contribution as well. In fact, the high doses of 185 and 385 mg/kg/day provided to female humans for 90 days [37] elicited presence of steatorrhea, whose percentage of incidence was dose-dependent. Overall, doses and matrices of squalene administration may have a profound influence.

Another interesting aspect noted in WT mice receiving squalene was that the increase in HDL-cholesterol was mainly due to augmented esterified cholesterol (Fig. 1B). This fact indicates that the activity of the enzyme responsible for this type of cholesterol in HDL, lecithin∶cholesterol acyltransferase (LCAT), was increased. Elevated hepatic *Lcat* mRNA expression (Table 5) supported this finding. LCAT plays an important physiological role in the process of maturing HDLs, modulating the conversion of HDL_3_ to HDL_2_, and thereby collecting cholesterol from peripheral tissues and increasing HDL levels [44]. In these WT mice, HDL was enriched in phosphatidylcholine (PC) (Fig. 1C), which is the substrate for the LCAT activity [45], indicating that squalene is favoring the secretion of an HDL enriched in LCAT and with an appropriate endowment of PC to facilitate its action and thereby reverse cholesterol transport and without changes in the LCAT activators APOA1 or APOA4 [46]. Since secretion of LCAT is associated with APOA1 [47], these changes would not be possible in its absence, as in the case of *Apoa1*-deficient mice. In *Apoe*-deficient mice, it would not be possible either, due to the high content of remnant and intermediate density lipoproteins characteristics of this model [31]. For this reason, in these mice important amounts of APOA1 and APOA4 were observed in VLDL and LDL (Fig. 1P and Q), suggesting incomplete liberation of nascent HDL from chylomicrons/VLDL according to the hypothesis of HDL secretion as part of those lipoparticles [48] and in agreement with the postprandial time follow-up of APOA4 distribution [49]. In this setting, squalene would promote a maturation of HDL by transferring these APOS to the HDL fraction. Overall, squalene is exerting important effects on these lipoproteins depending on their inherent metabolic characteristics present in the studied model.

The increase in HDL-cholesterol levels found in this study was also accompanied by a significant increase in serum arylesterase activity in WT and *Apoa1*-deficient mice consuming squalene, in HDL-PON1 and *Pon1* mRNA expression in WT mice and in HDL-PON1 in *Apoe*-deficient mice consuming 1 g/kg of squalene. Human PON1 is synthesized in the liver and secreted into the blood, where it is mainly associated with HDLs [50], hydrolyzes oxidized phospholipids and protects against the development of atherosclerosis [51], [52]. HDL's ability to prevent oxidative modifications of LDL has been attributed to PON1 [53], [54]. The observed rise in this enzyme activity could likely explain the decreased ROS content in VLDL, LDL and HDL together with PON2 due to the changes observed for *Pon2* and *Pon1* in WT mice. In the case of *Apoa1*-deficient mice, no changes were found either in HDL-PON1 or in hepatic *Pon1* expression, but *Pon2* and *Pcyox1* mRNA were significantly decreased (data not shown), so increased serum arylesterase activity and decreased *Pcyox1* in liver could explain the decrease in LDL- and HDL-ROS content of mice lacking *Apoa1* consuming squalene. In the latter mice, HDL-PON1 was not changed and together with absence of APOA1, the changes induced by squalene on HDL were inefficient to decrease MDA levels (Fig. 2E). On the other hand, when HDL-PON1 increased by squalene such as in WT and in *Apoe*-deficient mice receiving 1 g/kg, there were significant decreases in MDA levels. Overall, these results indicate that squalene is exerting an antioxidant action mediated by inducing PON1 content of HDL and that this action requires the presence of APOA1. In this regard, squalene behaves like compounds from pomegranate juice, which decreased oxidative stress in diabetic patients by increasing PON1 association with HDL and stimulating its enzyme activity [55], [56]. However, as squalene has been reported to own antioxidant properties as a quencher of singlet oxygen [57] and as an *in vitro* highly effective oxygen scavenging agent [12], a direct involvement of this molecule cannot be discarded. Furthermore, it has been reported to elicit antioxidant defense proteins [58]. Thus, the mechanisms whereby squalene protects against oxidative damage in lipoproteins need to be further studied.

A well-controlled study was conducted in elderly hypercholesterolemic patients using a 12 mg/kg/day dose of squalene for 20 weeks. Despite the fact that this dose alone did not modify plasma cholesterol levels, when combined with a low dose of pravastatin, it potentiated the hypolipidemic properties of the statin [59]. These data together with the described properties found in this work are promising aspects to go further and clarify some of the emerging caveats regarding dose, experimental model and administration matrices.

In conclusion, the increase in plasma HDL-cholesterol and PON1 levels and the decrease in ROS in VLDL, LDL and HDL seem to be the mechanisms whereby squalene exerts its beneficial activity, hence it could be a contributor to the beneficial effects of the virgin olive oil intake on the prevention against cardiovascular diseases. This squalene action is conditioned by the genetic background of APOA1 and APOE, characteristics that modulate the maturation's state of secreted HDL, the ability to secrete functional LCAT or the changes in HDL-PON1. In addition, formulation of dietary squalene in terms of dose and accompanying elements in the alimentary matrix should be carefully considered to facilitate reproducible results.

## Materials and Methods

### Animals

Male wild-type (WT) and *Apoe*-deficient mice on C57BL/6J genetic backgrounds were obtained from Charles River (Charles River Laboratories, Barcelona, Spain). *Apoa1*-deficient mice on C57BL/6J genetic background were generously provided by Dr. Nobuyo Maeda from University of North Carolina at Chapel Hill. To establish groups with similar initial plasma cholesterol, blood samples were taken from the facial vein after four-hour fasting. All animals were housed in sterile filter-top cages in rooms maintained under a 12-h light/12-h dark cycle in the *Servicio de Biomedicina y Biomateriales*, University of Zaragoza. All had *ad libitum* access to food and water and the study protocol was approved by the Ethics Committee for Animal Research of the University of Zaragoza.

### Diets

All male mice were fed chow semipurified diets and divided into control group (n = 6 for WT, n = 7 for *Apoa1*- and n = 13 for *Apoe*-deficient mice) and squalene-treated animals (n = 7 for both WT and *Apoa1*-deficient mice), these latter receiving 1 g squalene/kg body weight, except for the *Apoe*-deficient animals, in which squalene-treated animals were given two different doses of the compound: 0.25 (n = 13) or 1 g (n = 14) squalene/kg body weight. All diets were prepared weekly and stored in an N_2_ atmosphere at −20°C. Fresh food in excess was provided daily, and the differences of amount provided and the remaining per cage were recorded daily, divided into number of animals and used to assess the average food intake. The animals were fed the experimental diets for 11 weeks, being well tolerated.

### Biochemical determinations

After the experimental period, animals were sacrificed by suffocation with CO_2_, blood (0, 7–1 ml per mouse) was collected via cardiac puncture and centrifuged at 3000 rpm for 5 minutes at 4°C to obtain plasma. Total plasma cholesterol and triglyceride concentrations were measured in a microtitre assay, using Infinity commercial kits (Thermo Scientific Madrid, Spain). Plasma HDL-cholesterol was quantified in the supernatant after precipitation of apoB particles with phosphotungstic acid–MgCl_2_ (Roche, Barcelona, Spain). Glucose and non-esterified fatty acids were determined using kits from BioSystems (Barcelona, Spain) and Wako (Madrid, Spain). Paraoxonase was assayed as arylesterase activity by the rate of phenylacetate hydrolysis, as described previously [60]. APOA1 and APOA4 were quantified by ELISA using specific polyclonal antibodies (Biodesign and Santa Cruz Biotechnology), as described previously [61]. Malondialdehyde was assayed following Conti's spectrofluorimetric method [62]. Plasma lipoprotein profile was determined in 100 µl of pooled plasma samples from each group by fast protein liquid chromatography (FPLC) gel filtration [63] using a Superose 6B column (GE Healthcare), and the cholesterol, phosphatidylcholine and sphingomyelin contents in each fraction were measured as described [49].

### *Reactive oxygen species (ROS) content in* lipoproteins

The presence of ROS was assessed by measuring the conversion of 2,7-dichlorofluorescein diacetate into fluorescent dichlorofluorescein [64] in FPLC-isolated fractions corresponding to the different lipoproteins [65]. Briefly, LDL or VLDL (0.8 µg cholesterol for wild-type and *Apoa1*-deficient mice, and 1.5 µg cholesterol for *Apoe*-deficient mice) and HDL (60 ng cholesterol) were incubated at 37°C with 2 µg dichlorofluorescein in 2.5 µl of 0.1% sodium azide and PBS up to a total volume of 240 µl. After 24 h of incubation, fluorescence was measured at an excitation wavelength of 485 nm and an emission wavelength of 535 nm [65]. To study the protective action of HDL against oxidation, LDL particles were incubated in the presence of its HDL, and fluorescence was measured.

### Western blotting

HDL fractions (30 µg of protein) were loaded onto 12% SDS-PAGE gels, electrophoresed, and transferred as previously described [25]. Protein bands were detected using a rabbit polyclonal anti-PON1 antibody raised against a mouse oligopeptide (CYKNHRSSYQTRLNAFREVTP) at dilution 1/1,000 [66]. As secondary antibody, a donkey anti-rabbit conjugated with IRdye 680RD (926-68073 LI-COR Biosciences, Lincoln, NE, USA) at 1/15,000 dilution was used. Image was captured and analyzed using an Odyssey Clx (LI-COR).

### RNA isolation

At sacrifice, the livers were immediately removed and frozen in liquid nitrogen. RNA from each liver was isolated using Tri-reagent (Ambion, Austin, TX, USA). DNA contaminants were removed by TURBO DNAse treatment using the DNA removal kit from Ambion. RNA was quantified by absorbance at A_260/280_. The integrity of the 28 S and 18 S ribosomal RNAs was verified by agarose gel electrophoresis.

### Quantification of mRNA

The differences in mRNA expression of genes involved in cholesterol metabolism or antioxidant defense were studied by quantitative real-time RT-qPCR analysis using equal amounts of DNA-free RNA from each animal. First-strand cDNA synthesis was generated using the First Strand Synthesis kit (Thermo Scientific, Madrid, Spain). Reverse transcriptase-quantitative polymerase chain reactions (RT-qPCR) were performed using the Sybr Green PCR Master Mix (Applied Biosystems, Foster City, CA, USA). The primers were designed using Primer Express (Applied Biosystems) and checked by BLAST analysis (NCBI) to verify gene specificity as well as to get amplification of the cDNA and not of genomic DNA. The sequences were published [67]–[69]. Real time RT-qPCR reactions were carried out in a Step One Real Time PCR System (Applied Biosystems) following the standard procedure. The relative amount of all mRNAs was calculated using the comparative 2^−ΔΔCt^ method and normalized to the reference *Cyclophilin B*, *Cyclophilin A* and TATA box binding protein, *Tbp* mRNA expressions [68].

### Statistical analysis

Results are expressed as means ± SD. Unless otherwise stated, non parametric Mann-Whitney U-test for comparison between pairs was used for unpaired observations. Association between variables was assessed by Spearman correlation test. All statistical tests were performed with the package SPSS version 15.0 (SPSS Inc, Chicago, IL, USA), and a value of p<0.05 was considered statistically significant.
